# Associations among perceived negative consequences, perceived deterioration in well-being, fear of progression, and probable depression in Chinese adults with chronic kidney disease: a cross-sectional path analysis

**DOI:** 10.3389/fpsyg.2026.1779503

**Published:** 2026-10-05

**Authors:** Tongxu Zhang, Ting Fang, Xueyi Li, Yun Wu

**Affiliations:** 1 The Second People’s Hospital of Lishui, Wenzhou Medical University, Lishui, Zhejiang, China; 2 The Third People’s Hospital of Qujing, Qujing, Yunnan, China

**Keywords:** chronic kidney disease, fear of progression, perceived deterioration in well-being, perceived negative consequences, probable depression

## Abstract

**Introduction:**

Individuals with chronic kidney disease (CKD) are vulnerable to probable depression. Perceived negative consequences (PNC) of CKD, perceived deterioration in well-being (PDW), and fear of progression (FoP) are commonly experienced in this population. This cross-sectional study examined the associations among PNC, PDW, FoP, and probable depression. It also tested the indirect associations of PNC and PDW with probable depression through FoP. Additionally, gender differences in these direct and indirect pathways were explored.

**Methods:**

In this cross-sectional study, a total of 456 Chinese CKD patients were recruited using convenience sampling. Participants completed standardized self-report questionnaires at a single time point. Path analysis and multi-group analysis by gender were conducted using Mplus 8.3.

**Results:**

The prevalence of probable depression was 37.3%. PDW, PNC, and FoP were positively and significantly associated with probable depression. Significant indirect associations of both PDW and PNC with probable depression through FoP were observed. Significant gender differences were found in the PDW → FoP, PNC → FoP, and PDW → probable depression associations, as well as in the indirect associations of both PDW and PNC with probable depression through FoP. All these associations were stronger among females than males. No significant gender differences were observed in the FoP → probable depression or PNC → probable depression associations.

**Conclusion:**

Higher levels of PNC, PDW, and FoP were associated with a greater likelihood of probable depression among individuals with CKD. The significant indirect associations through FoP were consistent with its potential explanatory role in the proposed model. Longitudinal and intervention studies are needed to determine whether addressing illness appraisals or FoP can improve psychological well-being in this population.

## 1 Introduction

Chronic kidney disease (CKD) is a globally prevalent condition characterized by structural or functional abnormalities of the kidneys lasting for at least 3 months (Bahall et al., 2023). Epidemiological studies estimate that CKD affects approximately 9.5% of the global adult population (Jadoul et al., 2024). Patients with CKD frequently experience psychological distress, including anxiety and probable depression (Peng et al., 2015). For example, in a meta-analysis of CKD populations, the pooled prevalence of probable depression was 26.5% (95% CI: 23.1%–30.1%) (Adejumo et al., 2024). Another meta-analysis reported a similarly high pooled prevalence (21.4%–39.3%) across CKD stages (Palmer et al., 2013). Empirical studies conducted in different cohorts have reported comparable findings, with probable depression observed in 22.2%–60.3% of kidney disease patients across regions (Duan et al., 2021; Bahall et al., 2023; Chilcot et al., 2025). Probable depression in CKD patients has been associated with poorer treatment adherence, more rapid renal deterioration, and higher mortality (Palmer et al., 2013; Adejumo et al., 2024). Given the high prevalence of probable depression and its adverse impact among CKD patients, it is imperative to investigate factors associated with probable depression in this population.

Perceived negative consequences (PNC) refers to an individual’s beliefs about the extent to which an illness disrupts their life, health, and social functioning (Moss-Morris et al., 2002). Within the framework of the Common-Sense Model (Leventhal et al., 2016), such perceptions constitute a key component of illness representations and have been shown to be associated with emotional outcomes. Patients who perceive their illness as having severe and pervasive consequences are more likely to experience psychological distress, including depression (Kapetanakis et al., 2023; Jaltuszewska et al., 2023; Al-Sutari and Khraisat, 2025). In chronic illness populations such as CKD, higher perceived illness consequences have been linked to greater depressive symptom severity and poorer adjustment (Muscat et al., 2021; Keskindag et al., 2025). Given that CKD often involves fluctuating symptoms, treatment burden, and lifestyle limitations, patients may develop strong negative appraisals regarding its impact on daily life and health. These negative perceptions may be accompanied by feelings of hopelessness and helplessness and by more severe depression (Cengiz and Çıtlık Sarıtaş, 2019; Li et al., 2025). Understanding how PNC are associated with emotional adjustment among CKD patients is therefore crucial for identifying those with greater psychological distress and for informing future longitudinal and intervention research.

Perceived deterioration in well-being (PDW) refers to an individual’s subjective assessment of whether their current health has deteriorated relative to their pre-illness status (Wang et al., 2020). In the present study, PDW was operationalized as a comparative assessment of current physical and psychological health relative to pre-illness status. Thus, unlike PNC, PDW is defined by a temporal comparison with a pre-illness baseline rather than by the perceived breadth or severity of CKD-related consequences across life domains. In chronic and infectious disease contexts, such perceptions often coexist with negative illness beliefs and have been linked to heightened psychological distress, particularly depression. For example, perceived declines in health and functional status have been shown to be associated with later probable depression (Leinonen et al., 2001). Recent studies during the COVID-19 pandemic similarly reported that individuals perceiving deterioration in their physical or psychological health were significantly more likely to experience anxiety and depression (Wang et al., 2020; Tur-Sinai et al., 2022). These studies indicate that perceived worsening of health is associated with feelings of helplessness and loss of control, as well as with probable depression (Jaltuszewska et al., 2023). In the context of CKD, persistent fatigue, edema, or reduced renal function may coexist with poorer perceived health and greater probable depression (Tanemoto et al., 2023). Examining the association between PDW and probable depression may thus provide valuable insights into their psychological correlates and potential relevance to patient care.

Individuals with CKD may experience fear of progression (FoP), which refers to the persistent fear that their disease may worsen, recur, or lead to further physical, psychological, or social consequences (Sharpe et al., 2023a; Xu et al., 2024). This fear has been strongly associated with adverse mental health outcomes, particularly anxiety and depression (Sharpe et al., 2023b; Xu et al., 2024). Theoretically, FoP is an emotional representation within the CSM framework. According to the CSM of illness, an illness is a stressor resulting in formation of cognitive and emotional illness representations, both of which would in turn be associated with an individual’s health-related outcomes, including mental distress (Leventhal et al., 2016). Empirically, unfavorable perceptions of illness consequences have been shown to be positively associated with probable depression (Lu et al., 2024a; Al-Sutari and Khraisat, 2025). Specifically, PNC and PDW have been linked to emotional responses like FoP. Therefore, FoP may be involved in indirect associations of PNC and PDW with probable depression. For instance, persistent symptoms in CKD are often associated with FoP, which in turn significantly correlates with probable depression. This aligns with the proposition that illness-related stressors are associated with prolonged fear, which is in turn associated with adverse mental health outcomes (Brosschot et al., 2006). Thus, under the CSM framework, FoP may represent a potential explanatory correlate for the observed illness-related appraisals and probable depression. Clarifying these associations may inform future longitudinal and intervention studies among patients with CKD.

Emerging evidence suggests that the associations among PNC, PDW, FoP, and probable depression may differ between females and males. In chronic illness populations, females generally report higher levels of psychological distress and perceive greater health deterioration than males (Um-E-Kalsoom, 2020). Studies among patients with CKD have similarly found that females are more likely to experience depressive symptoms, fatigue, and illness-related anxiety (Um-E-Kalsoom, 2020; Bahall et al., 2023). However, the factors underlying these differences remain uncertain, and whether the associations among illness appraisals, FoP, and probable depression differ between females and males has not been established. These observations provide a rationale for examining whether the magnitudes of the associations among illness perceptions, FoP, and probable depression differ between females and males with CKD.

The present study aimed to examine the prevalence of probable depression among Chinese adults with CKD. Specifically, it investigated the associations of PNC, PDW, and FoP with probable depression. Drawing upon the CSM, this study evaluated a hypothesized model in which PNC and PDW were associated with probable depression both directly and indirectly through FoP. In addition, given prior evidence of gender-related differences, the study further explored whether the magnitudes of these associations differed by gender. Accordingly, three hypotheses were proposed: (1) PNC, PDW, and FoP would be positively associated with probable depression; (2) PNC and PDW would show significant indirect associations with probable depression through FoP; and (3) the magnitudes of the direct and indirect associations would differ by gender.

## 2 Materials and methods

### 2.1 Participants and data collection

Participants in this study were adult patients clinically diagnosed with chronic kidney disease (CKD) who were recruited from three hospitals in Zhejiang, China, between June and December 2024. Patients were eligible to participate if they had a confirmed diagnosis of CKD based on established clinical criteria or laboratory evidence, including an estimated glomerular filtration rate (eGFR) below 60 mL/min/1.73 m^2^ and/or markers of kidney damage (e.g., Urine Albumin-to-Creatinine Ratio ≥30 mg/g or abnormal renal imaging) persisting for at least 3 months. Additional inclusion criteria were: aged 18 years or older, able to read and understand Chinese, and willing to provide written informed consent.

Patients were excluded if they had severe psychiatric disorders (e.g., schizophrenia or bipolar disorder), acute kidney injury, end-stage renal disease requiring dialysis or kidney transplantation, significant cognitive impairment, or were currently hospitalized for acute complications. Eligible participants were approached by nephrology clinicians during routine outpatient visits or inpatient care and were invited to participate voluntarily. After receiving a full explanation of the study, each participant signed a written informed consent form and completed an anonymous paper-based questionnaire, which required approximately 15 min.

In total, 475 participants completed the survey. After excluding incomplete or invalid responses (i.e., questionnaires with less than 90% completion or evident response patterns), 456 valid questionnaires were retained for analysis. Ethical approval was obtained from the Ethics Committee of the corresponding author’s institution (Approval No. 20241205–01), and all study procedures were conducted in accordance with the Declaration of Helsinki.

### 2.2 Measurement

#### 2.2.1 Background factors

Information about participants’ gender, age, community type, educational level, marital status, employment status, CKD stage, and chronic disease status was collected. Gender was self-reported using male and female response categories.

#### 2.2.2 Probable depression

The 9-item Patient Health Questionnaire (PHQ-9) was used to assess probable depression (Kroenke et al., 2001). Participants rated how often they had experienced each symptom over the past 2 weeks. Sample items include “little interest or pleasure in doing things” and “feeling down, depressed, or hopeless.” Responses were recorded on a four-point Likert scale ranging from 0 (not at all) to 3 (nearly every day). Total scores range from 0 to 27, with a cutoff point ≥10 indicating probable depression. This predefined threshold was selected because it has demonstrated a favorable balance between sensitivity and specificity in the original validation study and subsequent diagnostic-accuracy meta-analyses (Kroenke et al., 2001; Manea et al., 2012; Levis et al., 2019; Negeri et al., 2021). The same threshold has also demonstrated validity in dialysis patients and Chinese clinical populations (Watnick et al., 2005; Xiong et al., 2015) and has been used to define probable depression in previous studies (Wang et al., 2021; Lu et al., 2024a). Because the PHQ-9 is a screening instrument rather than a diagnostic interview, participants who scored ≥10 were classified as having probable depression rather than clinically diagnosed depression. It has been validated and showed excellent psychometric properties among Chinese adults (Lu et al., 2024a). The Cronbach’s α was 0.92 in this study.

#### 2.2.3 Perceived negative consequences (PNC)

Perceived negative consequences were assessed using the Consequences subscale of the Illness Perception Questionnaire–Revised (Moss-Morris et al., 2002). This subscale measures the extent to which patients believe their illness has adverse effects on various aspects of their lives, including physical health, emotions, daily functioning, and social relationships. It consists of six items, each rated on a five-point Likert scale ranging from 1 (strongly disagree) to 5 (strongly agree). Higher scores indicate stronger perceptions of the illness as having negative and pervasive consequences. The Cronbach’s α was 0.86 in this study.

#### 2.2.4 Perceived deterioration in well-being (PDW)

Perceived deterioration in well-being was evaluated using two items adapted from the comparative self-perceived health questionnaire developed by Wang et al. (2020). The original assessment evaluated perceived changes in physical and psychological health by comparing health ratings across two reference periods. Because the present study focused on perceived deterioration following CKD, pre-illness health was used as the reference, and participants directly compared their current physical and psychological health with their pre-illness status. The two items are: “How is the state of your physical health compared with your pre-illness status?” and “How is the state of your psychological health compared with your pre-illness status?”. The rating for each question ranged from 1 (much better), 2 (mildly better), 3 (the same), 4 (mildly worse), and 5 (much worse), with higher scores indicating greater PDW. The Cronbach’s α was 0.85 in this study.

#### 2.2.5 Fear of progression (FoP)

Fear of progression was assessed using the Fear of Progression Questionnaire-Short Form (Mehnert et al., 2006). The FoP-Q-SF consists of 12 items that evaluate patients’ fears and worries about disease worsening and its potential physical, psychological, and social consequences. Each item is rated on a five-point Likert scale ranging from 1 (never) to 5 (very often), with higher scores indicating greater fear of disease progression. The total score is obtained by summing all item responses, with higher values representing higher levels of FoP. In the present study, the validated Chinese version of the FoP-Q-SF was used (Ma et al., 2023). The Cronbach’s α was 0.91 in this study.

### 2.3 Statistical analysis

Probable depression was operationalized as a binary outcome variable (0 = PHQ-9 score <10; 1 = PHQ-9 score ≥ 10) in all analyses. Descriptive analyses were first conducted to summarize participants’ demographic characteristics. Univariate logistic regression analyses were performed to test the associations between background factors and probable depression. Because all main variables were assessed using self-report questionnaires at a single time point, potential common method bias was evaluated using Harman’s single-factor test. All items measuring PNC, PDW, FoP, and probable depression were entered simultaneously into an unrotated factor analysis. The variance explained by the first factor was examined to determine whether a single factor dominated the covariance among the study measures. Separate multivariate logistic regression analyses were conducted to assess the relationships of PNC, PDW, and FoP with probable depression. For each variable, an unadjusted model, a partially adjusted model, and a fully adjusted model were fitted. The partially adjusted model included background factors which were significantly associated with probable depression in the univariate analyses. The fully adjusted model included all measured background factors considered potentially relevant to probable depression.

Path analyses were performed to examine the indirect associations of PNC and PDW with probable depression through FoP. A partially adjusted path model and a fully adjusted path model were estimated using the same two sets of covariates described above, respectively. The Weighted Least Squares Mean and Variance Adjusted (WLSMV) estimator was applied. Satisfactory model fit indices included Comparative Fit Index (CFI) ≥ 0.90, Tucker-Lewis Index (TLI) ≥ 0.90, Root Mean Square Error of Approximation (RMSEA) ≤ 0.08, and Standardized Root Mean Square Residual (SRMR) ≤ 0.08 (Kline, 2016). Multi-group analysis was then performed to examine the gender difference in the significance of the mediation model’s structural/indirect paths. An unconstrained baseline model was first estimated, in which all structural paths were freely estimated across gender. Subsequently, a series of nested models were tested by constraining each structural or indirect path to be equal across gender. Standardized coefficients were reported. Wald χ^2^ tests were used to compare the constrained and unconstrained models; a significant Wald test (*p* < 0.05) indicated a statistically significant gender difference in the corresponding path, whereas a non-significant result suggested no evidence of a gender difference.

To address the potential conceptual overlap between the psychological-health item of PDW and probable depression, we conducted sensitivity analyses using only the physical-health item, operationalized as perceived physical health deterioration (PPHD). First, the association between PPHD and probable depression was examined using separate binary logistic regression models with the same covariate-adjustment strategy as that used in the primary analyses, including unadjusted, partially adjusted, and fully adjusted models. Second, the path model was re-estimated by replacing PDW with PPHD while retaining PNC, FoP, and probable depression. The direct association between PPHD and probable depression and its indirect association through FoP were examined to determine whether the principal findings remained robust after excluding the psychological-health item.

The path analysis and multi-group analysis were conducted using Mplus 8.3; the other analyses were performed by using SPSS 26.0. Statistical significance was defined as a two-tailed *p*-value < 0.05.

## 3 Results

### 3.1 Participants’ characteristics

As shown in Table 1, the mean (SD) age of the participants was 36.19 (10.14) years and 58.1% were female. Nearly half (48.0%) resided in urban areas, 62.7% were married, and 76.8% were employed full-time. Approximately 24.3% had attained a college degree or higher. In terms of health status, 37.3% reported having at least one chronic disease, and participants at CKD stages G3-G5 accounted for 49.6% of the sample. The prevalence of probable depression among individuals with CKD was 37.3%.

**TABLE 1:** Participants’ characteristics and associations with probable depression.

Variable	n/mean	%/SD	ORu	95% CI
Age	36.19	10.14	1.07	(1.05, 1.09)***
Gender				
Male	191	41.9	Ref	
Female	265	58.1	1.98	(1.33, 2.94)***
Community type				
Rural area	237	52.0	Ref	(0.77, 4.83)
City	219	48.0	1.92	
Educational level				
Middle school or below	161	35.3	Ref	(1.72, 4.19)***
High school	184	40.4	2.68	
College or above	111	24.3	0.65	(0.37, 1.14)
Marital status				
Married	286	62.7	Ref	(0.85, 1.85)
Others	170	37.3	1.25	
Employment status				
Fulltime	350	76.8	Ref	(0.50, 1.24)
Others (part-time, retired, homemaker, students)	106	23.2	0.79	
Chronic disease status				
No	286	62.7	Ref	(1.47, 3.23)***
Yes	170	37.3	2.18	
CKD stage				
G1	125	27.4	Ref	(0.36, 1.24)
G2	105	23.0	0.67	
G3	89	19.5	2.39	(1.35, 4.24)**
G4	82	18.0	2.95	(1.64, 5.30)***
G5	55	12.1	3.21	(1.66, 6.22)***
Probable depression				
No	286	62.7	N.A.	N.A.
Yes (PHQ-9 ≥ 10)	170	37.3	N.A.	N.A.

CKD, chronic kidney disease; Ref, reference group; N.A., not applicable. **p < 0.01; ***p < 0.001.

### 3.2 Background factors of probable depression

Age (ORu = 1.07) was significantly associated with probable depression. Gender (reference group = male, female: ORu = 1.98), educational level (reference group = middle school or below, high school: ORu = 2.68), whether having chronic disease status (reference group = no, yes, ORu = 2.18), and CKD stage (reference group = G1, G3: ORu = 2.39; G4: ORu = 2.95; G5: ORu = 3.21). Such information is presented in Table 1.

### 3.3 Assessment of common method bias

Harman’s single-factor test showed that the first unrotated factor accounted for 27.84% of the total variance. Thus, the data did not exhibit a dominant single-factor structure, suggesting that severe common method bias was unlikely.

### 3.4 Adjusted associations of PNC, PDW, and FoP with probable depression

As shown in Table 2, the separate multivariable logistic regression analyses showed that PNC (ORa = 1.55, 95% CI: 1.29–1.86), PDW (ORa = 2.04, 95% CI: 1.63–2.55), and FoP (ORa = 1.22, 95% CI: 1.16–1.28) were positively associated with probable depression in the partially adjusted models (all *p* < 0.001). These associations remained significant and were highly similar in magnitude in the fully adjusted models, with adjusted ORs of 1.54 (95% CI: 1.28–1.85) for PNC, 1.99 (95% CI: 1.61–2.45) for PDW, and 1.22 (95% CI: 1.16–1.28) for FoP (all *p* < 0.001).

**TABLE 2:** Associations between the independent variables and probable depression.

Variable	Unadjusted model		Partially adjusted model^$^		Fully adjusted model^#^	
	ORu	95% CI	ORa	95% CI	ORa	95% CI
PNC	1.57	(1.30, 1.89)***	1.55	(1.29, 1.86)***	1.54	(1.28, 1.85)***
PDW	1.94	(1.60, 2.36)***	2.04	(1.63, 2.55)***	1.99	(1.61, 2.45)***
FoP	1.23	(1.18, 1.28)***	1.22	(1.16, 1.28)***	1.22	(1.16, 1.28)***

^$^Partially adjusted for age, gender, educational level, chronic disease status, and CKD stage. ^#^Fully adjusted for age, gender, community type, educational level, marital status, employment status, chronic disease status, and CKD stage. PNC, perceived negative consequences; PDW, perceived deterioration in well-being; FoP, fear of progression. ***p < 0.001.

### 3.5 Path analysis

Figure 1 presents the results of the path analysis. The model fit indices were satisfactory (CFI = 0.974; TLI = 0.945; RMSEA = 0.043; SRMR = 0.037). PDW was positively associated with FoP (β = 0.40, *p* < 0.001), which was in turn positively associated with probable depression (β = 0.35, *p* < 0.001). The direct association of PDW on probable depression remained statistically significant (β = 0.09, *p* = 0.029). The indirect association between PDW and probable depression through FoP was significant (β = 0.14, SE = 0.03, 95% CI = [0.08, 0.20], *p* < 0.001), accounting for 60.9% of the total estimated association. Similarly, PNC was positively associated with FoP (β = 0.36, *p* < 0.001), which in turn was positively associated with higher probable depression (β = 0.35, *p* < 0.001). The direct association of PNC on probable depression was statistically significant (β = 0.15, *p* = 0.004). The indirect association between PNC and probable depression through FoP was also significant (β = 0.13, SE = 0.03, 95% CI = [0.07, 0.19], *p* < 0.001), which explained approximately 46.4% of the total estimated association.

**FIGURE 1:**
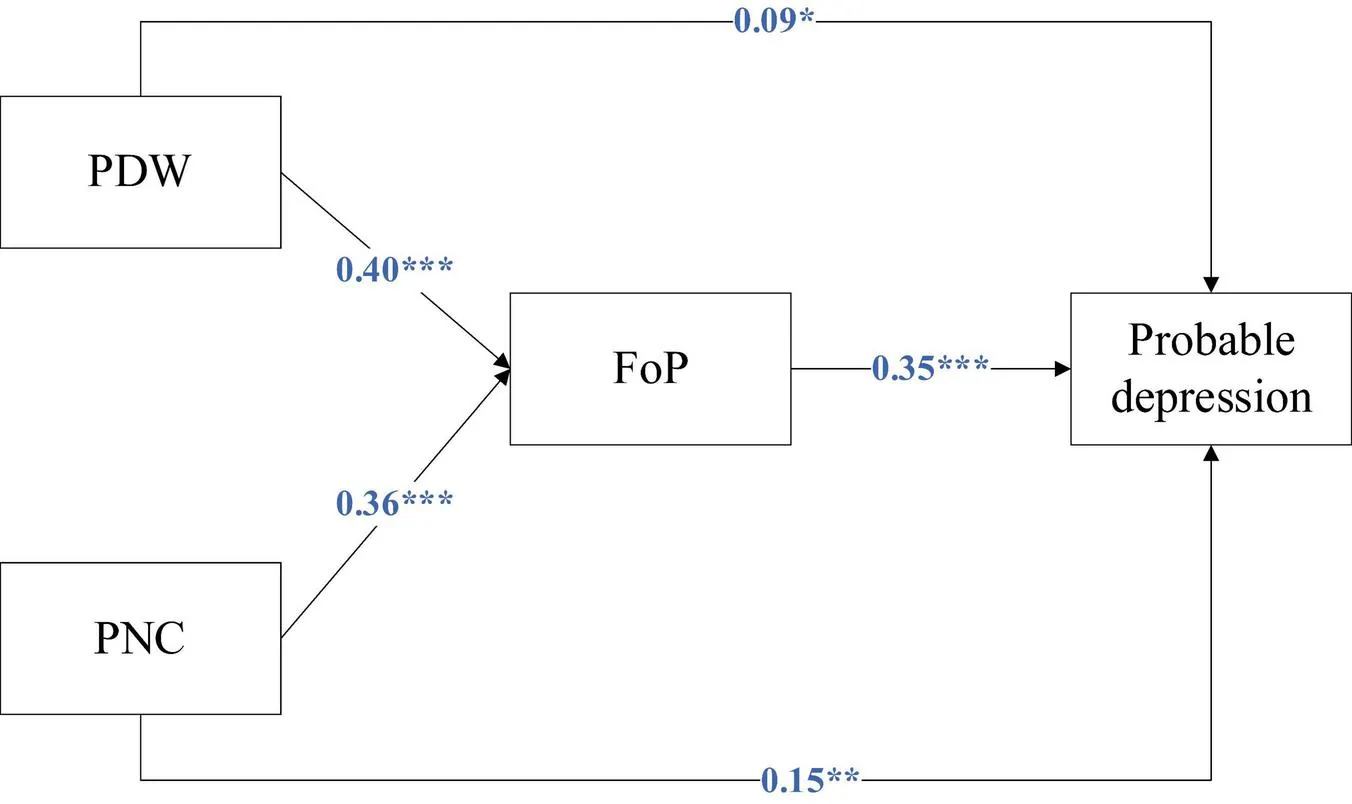
Partially adjusted path model of the indirect associations through FoP. Standardized coefficients (β) are shown (*N* = 456). The model was adjusted for age, gender, educational level, chronic disease status, and CKD stage; covariate paths are omitted for clarity. PDW, perceived deterioration in well-being; PNC, perceived negative consequences; FoP, fear of progression; PHQ-9, 9-item Patient Health Questionnaire. *p < 0.05; **p < 0.01; ***p < 0.001.

The path analysis was subsequently repeated after adjustment for all measured background factors. The fully adjusted model (Supplementary Figure 1) produced results that were highly similar to those of the partially adjusted model. PDW remained positively associated with FoP (β = 0.40, *p* < 0.001), while FoP remained positively associated with probable depression (β = 0.31, *p* < 0.001). The direct association between PDW and probable depression remained statistically significant (β = 0.10, *p* = 0.014), and its indirect association through FoP also remained significant (β = 0.12, SE = 0.02, 95% CI = [0.08, 0.16], *p* < 0.001), accounting for approximately 54.5% of the total association. Similarly, PNC remained positively associated with FoP (β = 0.36, *p* < 0.001). Its direct association with probable depression remained statistically significant (β = 0.14, *p* = 0.009), while its indirect association through FoP was also significant (β = 0.11, SE = 0.02, 95% CI = [0.07, 0.15], *p* < 0.001), accounting for approximately 44.0% of the total association. Thus, the pattern of direct and indirect associations remained consistent after adjustment for the full set of background factors, supporting the robustness of the findings.

### 3.6 Multi-group analysis by gender

The unconstrained baseline model demonstrated acceptable model fit, χ^2^(154) = 374.26, *p* < 0.001, CFI = 0.973, TLI = 0.962, RMSEA = 0.042, and SRMR = 0.029. A constrained model, in which structural paths were fixed to be equal across gender groups, also showed adequate fit to the data, χ^2^(159) = 423.66, *p* < 0.001, CFI = 0.958, TLI = 0.947, RMSEA = 0.058, SRMR = 0.043. Comparison of the constrained and unconstrained models indicated a significant deterioration in model fit when structural paths were constrained to be equal across gender, Δχ^2^(5) = 49.40, *p* < 0.001, suggesting that at least one of the constrained parameters differed significantly between the gender groups.

Gender differences in specific paths were subsequently evaluated using Wald χ^2^ tests of the equality constraints imposed on the corresponding path coefficients. The results are shown in Table 3. First, significant between-group differences were observed in the paths from PDW → FoP (Wald χ^2^ = 38.39, *p* < 0.001) and PNC → FoP (Wald χ^2^ = 8.43, *p* = 0.004); both associations were stronger among females than males. Second, a significant between-group difference was observed in the path from PDW → probable depression (Wald χ^2^ = 4.40, *p* = 0.036), with a stronger association observed among females. In contrast, the FoP → probable depression (Wald χ^2^ = 2.29, *p* = 0.130) and PNC → probable depression (Wald χ^2^ = 1.51, *p* = 0.219) paths did not differ significantly between the two groups. Third, significant between-group differences were also observed in the indirect associations of PNC → FoP → probable depression (Wald χ^2^ = 9.47, *p* = 0.002) and PDW → FoP → probable depression (Wald χ^2^ = 4.73, *p* = 0.030). Both indirect associations were statistically significant in males and females. However, their magnitudes were significantly greater among females than males for both the PDW → FoP → probable depression pathway (female: β = 0.20; male: β = 0.10) and the PNC → FoP → probable depression pathway (female: β = 0.16; male: β = 0.09).

**TABLE 3:** Multi-group analysis across gender.

Path	Male		Female		Wald test	
	β	*P*	β	*P*	*χ^2^*	*P*
Structural path						
(1) PDW → FoP	0.32	<0.001	0.55	<0.001	38.39	<0.001
(2) PNC → FoP	0.28	<0.001	0.44	<0.001	8.43	0.004
(3) FoP → probable depression	0.32	<0.001	0.36	<0.001	2.29	0.130
(4) PDW → probable depression	0.05	0.165	0.12	0.017	4.40	0.036
(5) PNC→ probable depression	0.07	0.346	0.18	0.003	1.51	0.219
Indirect path						
PDW→ FoP → probable depression	0.10	<0.001	0.20	<0.001	4.73	0.030
PNC→ FoP → probable depression	0.09	0.007	0.16	<0.001	9.47	0.002

PDW, perceived deterioration in well-being; PNC, perceived negative consequences; FoP, fear of progression.

### 3.7 Sensitivity analyses

To examine whether the findings involving PDW were driven by the potential conceptual overlap between its psychological-health item and probable depression, the analyses were repeated using PPHD. In the logistic regression analyses (Supplementary Table 1), higher PPHD was significantly associated with greater odds of probable depression in the partially adjusted model (ORa = 1.99, 95% CI: 1.61–2.45, *p* < 0.001) and the fully adjusted model (ORa = 1.98, 95% CI: 1.60–2.44, *p* < 0.001). In the partially adjusted path analysis (Supplementary Figure 2), PPHD remained positively associated with FoP (β = 0.34, *p* < 0.001) and probable depression (β = 0.11, *p* = 0.007). The indirect association between PPHD and probable depression through FoP also remained significant (β = 0.12, SE = 0.02, 95% CI = [0.08, 0.16], *p* < 0.001), while the remaining path coefficients were virtually unchanged. These findings indicate that the main associations remained robust after excluding the psychological-health item from the PDW measure.

## 4 Discussion

This study extends previous research by examining PNC, PDW, FoP, and probable depression within a single cross-sectional model among Chinese adults with CKD. The observed pattern was consistent with FoP as a potential explanatory correlate of the associations between illness appraisals and probable depression. Between-group heterogeneity was concentrated mainly in the associations of PNC and PDW with FoP and in the association between PDW and probable depression.

The prevalence of probable depression (37.3%) among individuals with CKD in this study falls within the higher range reported for CKD populations. Previous meta-analyses have estimated the pooled prevalence of probable depression in CKD at 21–39% (Palmer et al., 2013; Adejumo et al., 2024), while individual studies have reported rates between 22.2% and 60.3% (Duan et al., 2021; Bahall et al., 2023; Chilcot et al., 2025). Thus, the prevalence observed in the present study is broadly comparable to prior findings, though slightly higher than most estimates from non-dialysis CKD cohorts. Differences across studies may be related to variation in sample characteristics, disease severity, or treatment exposure, although these explanations were not examined in the present study. Overall, these findings suggest that depressive symptoms are highly prevalent in CKD and underscore the potential relevance of depressive symptom assessment in nephrology care.

Several background factors were associated with probable depression in CKD. Males had lower odds than females, aligning with evidence that females show higher rates of probable depression (Jensen et al., 2022). Younger patients showed lower odds, plausibly reflecting better renal reserve, fewer comorbidities, greater functional capacity, and more flexible social roles (Nablawi et al., 2025). The presence of additional chronic diseases was associated with higher odds of probable depression, consistent with multimorbidity research indicating biological load, polypharmacy effects, and activity limitations. Indicators of more severe CKD were linked to heavier depressive burden. This association may be related to fatigue, physical discomfort, body-image concerns, social withdrawal, or uncertainty regarding renal outcomes, although these factors were not directly assessed. These patterns are consistent with a biopsychosocial perspective in which biological, social, and cognitive factors are interrelated (Libório et al., 2012; Lin et al., 2021). These background factors may warrant consideration in future risk-prediction research, but prospective studies are required before they can be used to determine screening or referral strategies. The potential benefits of tailored communication, symptom management, and navigation support should be evaluated in intervention studies.

The positive associations between PNC, PDW, and probable depression are consistent with a relationship between illness appraisals and mood. PNC captures appraisals that CKD has harmful, pervasive effects on life domains, while PDW reflects a subjective sense of ongoing decline. Negative interpretations of symptoms, laboratory fluctuations, or treatment demands may coexist with demoralization and hopelessness. Findings from other chronic conditions echo this pattern: perceived functional loss and declining self-rated health have been longitudinally associated with subsequent probable depression and lower quality of life (Xiong et al., 2025; Leng et al., 2025). In CKD, persistent symptoms, treatment burden, dietary or fluid restrictions, and ongoing monitoring of kidney function may be accompanied by greater attention to symptoms, more negative illness beliefs, and greater depressive symptoms (Maher et al., 2025). However, the present findings cannot determine whether these appraisals precede, follow, or co-occur with probable depression. Accordingly, perceived consequences and deterioration may warrant evaluation as potential additions to psychosocial assessment alongside biological indicators. Intervention studies are needed to determine whether approaches that normalize symptom variability, reframe setbacks, and emphasize controllable behaviors can modify illness appraisals or depressive symptoms (Castro et al., 2021). Prospective longitudinal studies should test whether intentional cognitive change precedes and explains improvements in depressive symptoms and functioning across CKD stages.

A key finding was the significant indirect association between PDW and probable depression through FoP. This pattern accords with the CSM: cognitive representations (consequences) and emotional representations (fear) are conceptualized as interrelated components of illness representation (Leventhal et al., 2016). The findings also align with the perseverative cognition model, which proposes that persistent fear may be associated with sustained attention to health threats, physiological arousal, disrupted recovery behaviors, and low mood (Clancy et al., 2016). Clinically, FoP may represent a candidate for future assessment and intervention research. Previous interventions have addressed progression-related fear through psychoeducation about prognosis, normalization of symptom variability, activity planning, and exposure to avoided cues, alongside cognitive restructuring of interpretations (Burke et al., 2024). In nephrology settings, future studies could examine whether assessing FoP alongside symptom review provides clinically useful information. Future research should test temporal precedence, examining whether changes in FoP mediate symptom improvement when beliefs are targeted, and should also evaluate moderators such as CKD stage, symptom burden, renal function, and treatment status. If confirmed, interventions addressing FoP could be evaluated as a potential approach to improving mood, adherence, and quality of life in CKD.

Similarly, a significant indirect association between PNC and probable depression through FoP was observed. This finding is consistent with cognitive-behavioral models of illness adjustment, which propose relationships among catastrophic appraisals, threat perception, and emotional distress (Clark and Beck, 2023). Within this framework, exaggerated interpretations of bodily sensations or medical information are theorized to be associated with excessive worry, avoidance, and hopelessness. Interventions focusing on cognitive reframing and health literacy have been proposed as possible approaches for addressing negative interpretations of CKD (Magallón-Botaya et al., 2023; Tao et al., 2023). Whether enhancing perceived control, fostering realistic optimism, or promoting self-management reduces FoP or probable depression requires evaluation in intervention studies (Wang et al., 2025). Education sessions, motivational interviewing, and peer-support groups could similarly be evaluated as potential approaches to supporting resilience and agency. Overall, PNC-related cognitions may represent potential intervention targets, but their clinical relevance requires confirmation through longitudinal and intervention research in individuals living with CKD.

Between-group differences were not uniform across the model. They were observed in the PNC → FoP, PDW → FoP, and PDW → probable depression associations and in both indirect associations through FoP, but not in the FoP → probable depression or PNC → probable depression associations. Because the FoP → probable depression association was comparable across groups, the larger indirect associations among females appeared to reflect primarily the stronger associations of PNC and PDW with FoP. Thus, the principal between-group heterogeneity was located in the association between negative illness appraisals and progression-related fear rather than in the association between FoP and probable depression.

One potential explanation is that the subjective experience and appraisal of CKD-related health changes may differ between females and males. Women with advanced CKD have been reported to experience a greater subjective symptom burden despite having broadly comparable clinical profiles (van de Luijtgaarden et al., 2019), while studies in other chronic conditions have identified gender differences in perceptions of illness consequences, symptoms, controllability, and emotional impact (Grace et al., 2005; Bergsten et al., 2023). If perceived health changes or illness consequences had greater personal salience among the female participants, they may have shown a closer cross-sectional correspondence with concerns about future progression. A related possibility is that PDW, as an evaluation of changes in one’s current health, was more closely aligned with probable depression than the broader appraisal represented by PNC, which may help contextualize why a group difference was observed for the direct PDW path but not for the direct PNC path (Liu et al., 2021). However, symptom appraisal, personal salience, reporting processes, and relevant social or clinical characteristics were not directly assessed in this study. Therefore, these explanations should be regarded as hypotheses rather than conclusions. Future longitudinal and multimethod studies should determine whether the observed group differences can be replicated and whether they persist after accounting for objective disease severity, symptom burden, reporting tendencies, and gender-related social factors. Such research may help clarify whether the observed heterogeneity reflects differences in illness experience, illness appraisal, or measurement and reporting processes.

Results of the sensitivity analyses were broadly consistent with those of the primary analyses. After the psychological-health item was excluded, PPHD remained associated with probable depression in the logistic regression models and with both FoP and probable depression in the path model; an indirect association through FoP was also retained. Although the coefficient for the PPHD → FoP association was modestly smaller than the corresponding coefficient in the primary PDW model, the remaining path coefficients changed little. These findings lessen, but do not eliminate, the concern that the associations observed for PDW may have been influenced by conceptual overlap between its psychological-health item and probable depression.

The present findings have some potential implications for CKD care, although they should be interpreted cautiously given the cross-sectional observational design. The observed associations suggest that assessing PNC, PDW, and FoP may complement established probable depression screening by providing additional information about patients’ illness-related concerns and subjective health experiences. However, these factors should not be treated as diagnostic indicators or used independently for clinical risk stratification until their prospective predictive value has been established. Approaches such as psychoeducation, communication about prognosis and symptom fluctuations, cognitive-behavioral strategies, and multidisciplinary support may be relevant for addressing negative illness appraisals and progression-related fears (Davison et al., 2024; Lu et al., 2024b; Pearce et al., 2024). Nevertheless, the present findings do not demonstrate that modifying PNC, PDW, or FoP would reduce probable depression or improve adherence and quality of life. Similarly, the observed gender differences indicate possible heterogeneity in the associations among illness appraisals, FoP, and probable depression, but they do not provide sufficient evidence for gender-specific screening thresholds or intervention strategies. Longitudinal studies are needed to establish the temporal relationships among these variables and evaluate their predictive value. If these relationships are confirmed, intervention studies could then examine whether approaches targeting illness appraisals or FoP improve psychological outcomes. Subsequent implementation research would be required to assess the feasibility, acceptability, and cost-effectiveness of incorporating such approaches into routine CKD care.

Several limitations warrant consideration. First, the cross-sectional design precludes establishing temporal ordering or causal mediation. The direct and indirect associations are therefore consistent with the proposed model but cannot determine whether PNC or PDW preceded FoP and probable depression; reverse or reciprocal relationships remain possible. Second, all variables were self-reported at a single time point. Although Harman’s single-factor test did not indicate a dominant factor, it cannot exclude shared method variance, recall bias, social desirability, or mood-congruent reporting, which may have inflated the observed associations. The PHQ-9 identifies probable depression rather than a clinical diagnosis. Third, PDW was assessed using two items adapted from another health context. Although internally consistent, the measure captures only global perceived changes in physical and psychological health and has not been comprehensively validated in CKD. Its psychological-health item may also be conceptually related to the PHQ-9, and retrospective comparison with pre-illness health may be vulnerable to recall bias. The physical-only sensitivity analysis reduced, but did not eliminate, concern about this conceptual overlap. Further evaluation of convergent and discriminant validity, test–retest reliability, and responsiveness is needed. Fourth, convenience sampling from tertiary hospitals may limit generalizability to community-based patients and other regions. Finally, despite adjustment for all measured background factors, detailed information on disease duration, renal function, treatment status, and symptom burden was unavailable. Residual confounding by clinical severity therefore remains possible. Future multisite longitudinal studies should integrate repeated psychosocial assessments, clinical records, and diagnostic interviews.

## 5 Conclusion

This study identified a high prevalence of probable depression among Chinese CKD patients. Importantly, significant indirect associations of PNC and PDW with probable depression through FoP were observed, consistent with a potential explanatory role of illness-related fear within the proposed model. Furthermore, significant gender differences were observed, with females showing stronger associations of PNC and PDW with FoP and a stronger association between PDW and probable depression. These findings highlight patterns of association among illness appraisals, FoP, and probable depression. Clinically, illness appraisals and FoP may represent candidate targets for future intervention research, but the present findings cannot establish that modifying these factors would reduce probable depression. Future longitudinal and intervention studies are needed to establish temporal ordering and determine whether interventions addressing illness appraisals or FoP improve psychological outcomes in nephrology settings.

## Data Availability

The raw data supporting the conclusions of this article will be made available by the authors, without undue reservation.
