# Supplementary material for: Associations among perceived negative consequences, perceived deterioration in well-being, fear of progression, and probable depression in Chinese adults with chronic kidney disease: a cross-sectional path analysis
Source: Front Psychol. 2026 Oct 5;17:1779503. doi: 10.3389/fpsyg.2026.1779503 (PMC13677897; doi:10.3389/fpsyg.2026.1779503)
Supplement: Supplementary file 1 [file Table_1.docx]

Supplementary Figure 1 Fully adjusted path model of the indirect associations through FoP. Standardized coefficients (*β*) are shown (*N* = 456). The model was adjusted for all measured background factors: age, gender, community type, educational level, marital status, employment status, chronic disease status, and CKD stage. Covariate paths are omitted for clarity. PDW = perceived deterioration in well-being; PNC = perceived negative consequences; FoP = fear of progression; PHQ-9 = 9-item Patient Health Questionnaire.

*, *p* < .05; **, *p* < .01; ***, *p* < .001.

Supplementary Figure 2 Partially adjusted path model from the sensitivity analysis using the single-item PPHD measure. Standardized coefficients (*β*) are shown (*N* = 456). The model was adjusted for age, gender, educational level, chronic disease status, and CKD stage. Covariate paths are omitted for clarity. PPHD = perceived physical health deterioration, assessed using a single item comparing current physical health with pre-illness status; PNC = perceived negative consequences; FoP = fear of progression; PHQ-9 = 9-item Patient Health Questionnaire.

^*^, *p* < .05; ^**^, *p* < .01; ^***^, *p* < .001.
