# Supplementary material for: Associations among perceived negative consequences, perceived deterioration in well-being, fear of progression, and probable depression in Chinese adults with chronic kidney disease: a cross-sectional path analysis
Source: Front Psychol. 2026 Oct 5;17:1779503. doi: 10.3389/fpsyg.2026.1779503 (PMC13677897; doi:10.3389/fpsyg.2026.1779503)
Supplement: Supplementary file 2 [file Table_2.docx]

Supplementary Table 1 Association between perceived physical health deterioration (PPHD) and probable depression

| Variable | Unadjusted model | | Partially adjusted model $ | | Fully adjusted model # | |
| --- | --- | --- | --- | --- | --- | --- |
|  | ORu | 95% CI | ORa | 95% CI | ORa | 95% CI |
| PPHD | 1.86 | (1.53, 2.25) *** | 1.99 | (1.61, 2.45) *** | 1.98 | (1.60, 2.44) *** |

PPHD = perceived physical health deterioration; ORu = unadjusted odds ratio; ORa = adjusted odds ratio; CI = confidence interval. PPHD was assessed using the single item comparing participants’ current physical health with their pre-illness physical health, with higher scores indicating greater perceived deterioration.
^$^ Partially adjusted for age, gender, educational level, chronic disease status, and CKD stage.

^#^ Fully adjusted for age, gender, community type, educational level, marital status, employment status, chronic disease status, and CKD stage.

^*^, *p* < .05; ^**^, *p* < .01; ^***^, *p* < .001.
